# First person – Sarah Hoffmann

**DOI:** 10.1242/bio.040972

**Published:** 2019-01-15

## Abstract

First Person is a series of interviews with the first authors of a selection of papers published in Biology Open, helping early-career researchers promote themselves alongside their papers. Sarah Hoffmann is first author on ‘Three-dimensional movements of the pectoral fin during yaw turns in the Pacific spiny dogfish, *Squalus suckleyi*’, published in BiO. Sarah is a PhD Candidate in the lab of Dr Marianne Porter at Florida Atlantic University, USA, investigating the functionality of whole ecosystems.


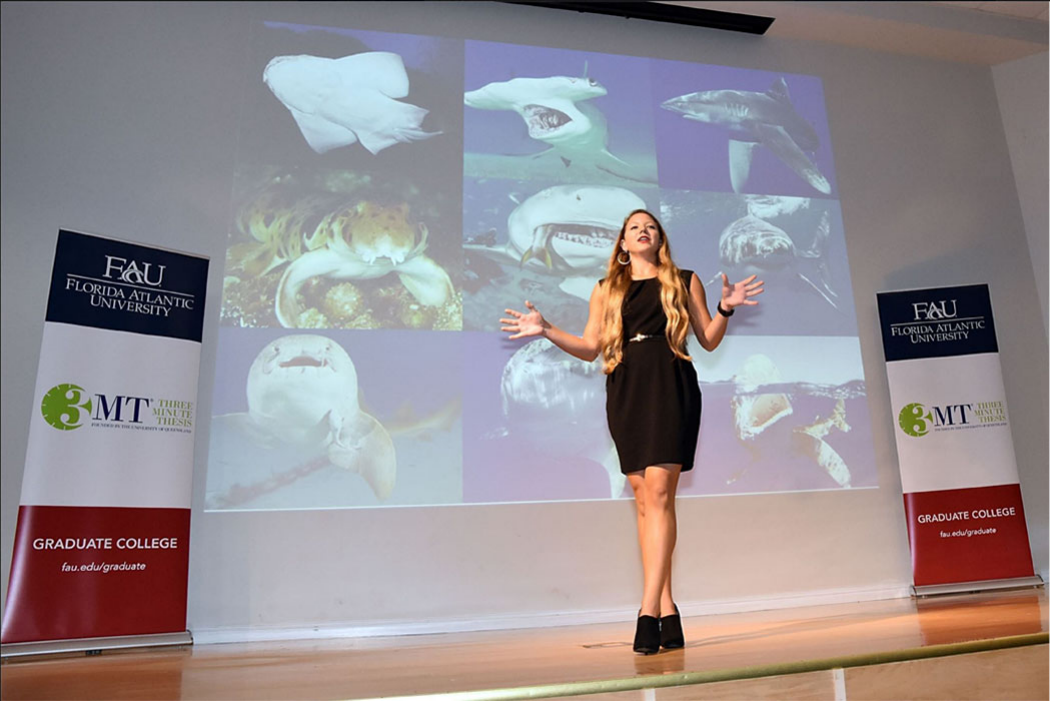


**Sarah Hoffmann**

**What is your scientific background and the general focus of your lab?**

Our lab focuses on biomechanics and biomaterials in swimming vertebrates. I am most interested the evolution of shape and function in different aquatic environments. My dissertation focuses on the comparative morphology and function of pectoral fins among sharks with varied whole-body morphology, locomotor style and habitat use.

**How would you explain the main findings of your paper to non-scientific family and friends?**

Pacific spiny dogfish have fins positioned near their mid-body that move substantially when they are turning. These sharks move their fins similar to the way kayakers use paddles to turn: the fin on one side of the body is rotated to create a pivot about which the body rotates.

**What are the potential implications of these results for your field of research?**

In this paper, we added to existing data demonstrating that shark pectoral fins are highly mobile control surfaces that play a major role in maneuvering. We also adapted motion capture techniques for use with underwater cameras in a relatively large environment, allowing us to capture 3D kinematics of free-swimming sharks. This technique was executed with low-cost, consumer grade cameras (GoPros) and free open source software, making it accessible to many researchers.

**What has surprised you the most while conducting your research?**

Working in the field and with live animals has taught me how effective low-cost solutions are. For example, our camera set-up in this study (for 3D video) was made entirely of cinderblocks, zip-ties and duct tape. I have been pleasantly surprised, and am a huge advocate for, the level of sophisticated meaningful research that can be done on a budget!

“I have been pleasantly surprised, and am a huge advocate for, the level of sophisticated meaningful research that can be done on a budget!”

**What, in your opinion, are some of the greatest achievements in your field and how has this influenced your research?**

I am most fascinated and influenced by advancements in the visualization of movement and morphology. The pioneering works of K. Liem and C. Gans, for example, have taught me to take integrative approaches to considering function while also remaining grounded in the anatomy of the organism. More recent advances in visualization such as XROMM, CT scanning, advanced microscopy and 3D particle image velocimetry have all revealed a new dimension to structure and function that we would have never accurately understood otherwise.

**What changes do you think could improve the professional lives of early-career scientists?**

In graduate school, I really struggled with work-life balance which I think is common among my peers. The competitive nature of grants and publishing drove me to prioritize work over all other aspects of life, and I missed out on a lot while I was in school. Recently, I've heard more conversations about maintaining balance and health in graduate school which I think is a good start. I am hopeful that the stereotype of the overworked, sleep-deprived, broken-spirited graduate student is changing.

“I am hopeful that the stereotype of the overworked, sleep-deprived, broken-spirited graduate student is changing.”

**What's next for you?**

I just accepted a position with Biomark, Inc. in Boise, ID, USA, as a fisheries scientist. Developing new techniques was the highlight of my dissertation work, and I am excited to be working on new designs and applications for wildlife tags. As technology advances, the level of precise detailed information we can get from tags makes looking to wildlife as ecosystem indicators a shrewd solution in our changing climate. I think that understanding the relationship between animal behavior, physiology and the environment will not only show the functionality of whole ecosystems, but will provide insight into more effective management practices moving forward.

**Figure:**
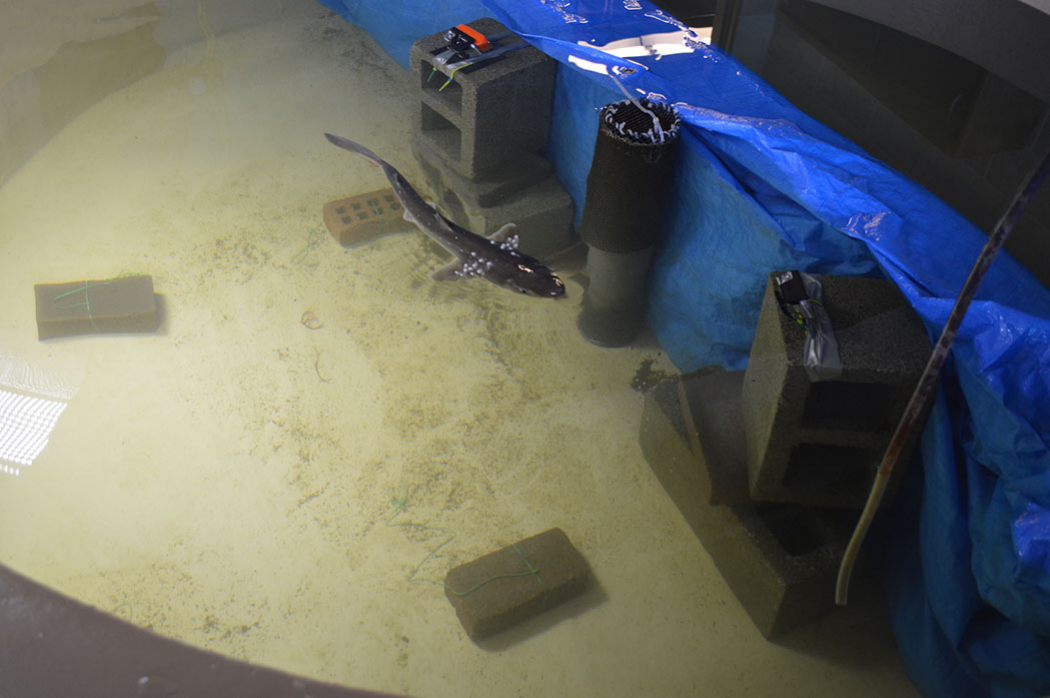
**A Pacific spiny dogfish outfitted with markers along the body and fin swims through a 3D calibrated volume for swimming kinematic analyses.**
